# A 3D MRI denoising algorithm based on Bayesian theory

**DOI:** 10.1186/s12938-017-0319-x

**Published:** 2017-02-07

**Authors:** Fabio Baselice, Giampaolo Ferraioli, Vito Pascazio

**Affiliations:** 1 Dipartimento di Ingegneria, University of Naples Parthenope, Centro Direzionale di Napoli, Is. C4, 80143 Naples, Italy; 2 Dipartimento di Scienze e Tecnologie, University of Naples Parthenope, Centro Direzionale di Napoli, Is. C4, 80143 Naples, Italy

**Keywords:** 3D MRI denoising, Maximum a posteriori, Markov random fields, Statistical signal processing

## Abstract

**Background:**

Within this manuscript a noise filtering technique for magnetic resonance image stack is presented. Magnetic resonance images are usually affected by artifacts and noise due to several reasons. Several denoising approaches have been proposed in literature, with different trade-off between computational complexity, regularization and noise reduction. Most of them is supervised, i.e. requires the set up of several parameters. A completely unsupervised approach could have a positive impact on the community.

**Results:**

The method exploits Markov random fields in order to implement a 3D maximum a posteriori estimator of the image. Due to the local nature of the considered model, the algorithm is able do adapt the smoothing intensity to the local characteristics of the images by analyzing the 3D neighborhood of each voxel. The effect is a combination of details preservation and noise reduction. The algorithm has been compared to other widely adopted denoising methodologies in MRI. Both simulated and real datasets have been considered for validation. Real datasets have been acquired at 1.5 and 3 T. The methodology is able to provide interesting results both in terms of noise reduction and edge preservation without any supervision.

**Conclusions:**

A novel method for regularizing 3D MR image stacks is presented. The approach exploits Markov random fields for locally adapt filter intensity. Compared to other widely adopted noise filters, the method has provided interesting results without requiring the tuning of any parameter by the user.

## Background

As most of clinical systems, Magnetic Resonance images are usually affected by artifacts and noise due to several reasons, such as the electronic noise generated by the thermal agitation of the charge carriers (thermal noise) and the imaging scanner technical limitations. Depending on the region of interest and on the application, noise could severely degrade the quality of acquired MR images and produce further errors in quantitative assessments from the data. Therefore, techniques for reducing the amount of noise affecting the acquisitions are usually implemented. In general, denoising methods aim at removing the undesirable noise while preserving the image details and local geometries [1].

One of the most used approaches for image denoising exploits the principle of averaging similar pixels of the image in order to lower the noise variance in the output image. This is mainly implemented in linear filters, such as Gaussian and boxcar. The main drawback is that the spatial average is equivalent to a low-pass filter in the frequency domain, thus image details and edges are degraded after the filter application. In other words, a blurring effect appears on the image [2].

To improve detail and edge preservation, nonlinear models have been proposed in literature [3, 4]. Anisotropic nonlinear diffusion belongs to this family of filters and has been developed for both regularizing the image and preserving edges [5]. The main drawback of this technique is the number of parameters that have to be tuned in order to reach effective performances.

Denoising can also be performed in transformed domains, where the separation between image and noise is expected to be easier. Of course, a proper transformation to the acquired data has to be applied. Among all, Wavelets are often adopted [6]. In case of MRI image, such approach introduces a bias in the filtered image. In order to reduce this disadvantage, the squared values of the image can be considered as the initial noisy one. However, such approach hardly preserve fine details of the images, especially in case of low signal to noise ratio (SNR). By considering as the transformed domain the spatial frequency one, Wiener filter has also to be taken into account [7, 8].

A different denoising approach consists in estimating the noise statistical parameters from the acquired images in order to reconstruct the noise-free image in a more effective way. To estimate noise characteristics, maximum likelihood estimation (MLE) can be implemented [9]. The main drawback of this approach is the assumption of constant signal in small regions, which produces poor details preservation.

Recently, a new filtering approach has been proposed, with very interesting detail preservation performances [2]. The methods assumes that there is redundancy across the image, and that similar patches can be found and jointly exploited for reducing noise. Such kind of methods, based on non local mean approaches [10], have proven to be very effective in images with high redundancy, but in some cases such as complicated structures or partial volume effect it fails, resulting in detail loss [11]. Another drawback of the technique is the required high computational burden.

In addition to previously reported ones, Markov random field (MRF) based methods provide interesting results [12, 13]. MRF exploits the spatial correlation information between a pixel and its neighborhood. This helps in reaching an estimation robust against noise and at the same time able to preserve fine structures and edges [14]. In other words, MRF allows a spatially adaptive noise regularization, able to reduce or increment signal smoothing according to the local statistical characteristics of the image. Often the implementation is iterative, making the approach computationally heavy.

Within this manuscript, a denoising technique based on MRF is exploited. In particular, following the approach of [14] developed for diffusion tensor MRI (DT-MRI), a maximum a posteriori (MAP) estimator is proposed for regularizing 3D amplitude MRI acquisition stacks. The peculiarity of the approach consists in defining a 3D local Gaussian MRF (LGMRF) that effectively adapts the model to the local behavior of the unknown image. In particular, with respect to a classic GMRF, this model considers a hyperparameters map that describes the spatial correlation between each pixel and its neighborhood. Such characteristic allows tuning the filter intensity, i.e. regularizing smooth areas while preserving edges and small details in an unsupervised way. In particular, if fine details are found a weak smoothing is applied in order to preserve them, while in case of flat areas a stronger regularization is implemented. The smoothing effect is automatically tuned by the MRF model in order to find the optimal trade-off between noise reduction and details preservation.

The manuscript is organized as follows: in the next Section the proposed methodology is presented, while in the following Section the framework for testing the performances of the approach is reported. Within the “Conclusion” section, the results are reported both in case of simulated and real datasets, together with a discussion about achievable performances compared to other widely adopted denoising methodologies. Finally, conclusions are drawn.

## Methods

The maximum a posteriori (MAP) estimator is defined via the so-called a posteriori distribution, which is the statistical description of the unknown parameters (in the considered case, the pixels of the noise free image stack *b*) after the data *a* has been acquired. Such probability density function is proportional to the product of the likelihood function and the a priori distribution:

1 $$\begin{aligned} f_{B}(b | a) \propto f_{A}(a| b) f_B(b) \end{aligned}$$

The likelihood function $f_{A}(a| b)$ is related to the statistical behavior of involved noise thus, in case of MR, it has a rice behavior [15–17]:

2 $$\begin{aligned} f_{A}(a | b) = \frac{a}{\sigma ^2} \exp { \left[ - \frac{(a^2+b^2)}{2 \sigma ^2} \right] } I_0 \left( \frac{ab}{\sigma ^2} \right) \end{aligned}$$

where $\sigma$ is the so called scale parameter and $I_0(\cdot )$ is the modified Bessel function of the first kind with order zero. The Rice distribution tends to a Gaussian one in case of high SNR (i.e. $b/ \sigma \rightarrow \infty$), and approaches a Rayleigh one in case of low SNR (i.e. $b/ \sigma \rightarrow 0$) [18]. In the framework of denoising applications, the low SNR case is more interesting and challenging, thus this assumption is made within this manuscript. In particular, considering the additive acquisition model:

3 $$\begin{aligned} a=b+n \end{aligned}$$

where *b* is the noise free image, which depends on the acquisition sequence [19], and *n* is the noise characterized by the following Rayleigh distribution:

4 $$\begin{aligned} f_{N}(n) = \frac{n}{\sigma ^2} \exp { \left[ - \frac{n^2}{2\sigma ^2} \right] } u \left( n \right) \end{aligned}$$

Combining Eqs. (3) and (4), the likelihood function related to the acquired data can be written as:

5 $$\begin{aligned} f_{A}(a | b) = \frac{(a-b)}{\sigma ^2} \exp { \left[ - \frac{(a-b)^2}{2\sigma ^2} \right] } u \left( a-b \right) \end{aligned}$$

where $u(\cdot )$ is the unit step function.

**Fig. 1:**
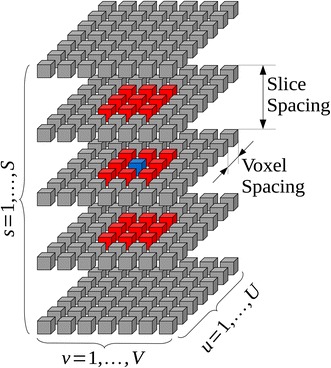
An example of 26-pixel neighborhood $\mathcal {N}_k$ (*red cubes*) for a selected location (*blue cube*) in a 3D stack of acquired images

The a priori distribution is derived from Markov random field theory. This is a widely adopted technique in image processing field [20–23]: it is able to model the statistical distribution of an image *b* taking into account the contextual dependencies of neighboring pixels. In other words, it is exploited for statistically describing the relationship between each pixel and its surroundings. Thanks to the Hammersley–Clifford theorem, an MRF can be analytically expressed in terms of Gibbs distribution [24]. This allows us to write the following expression for the a priori distribution:

6 $$\begin{aligned} f_B(b)=Z^{-1}\cdot \exp {\left[ -E(b, \theta )\right] } \end{aligned}$$

where $E(b,\theta )$ is the so-called *energy function* which depends on the image pixels and the hyperparameters $\theta$, while *Z* is a normalization parameter. In case of Local Gaussian MRF (LGMRF) the energy function can be defined as:

7 $$\begin{aligned} E(b,\theta )=\sum _{k=1}^{U \times V \times S}\sum _{q\in {\mathcal {N}_k}} \frac{(b_k-b_q)^2}{2\theta _{k,q}^2} \end{aligned}$$

where *U*, *V* and *S* are the number of rows, of columns and of slices of the acquired stack, respectively, while *q* is the index of pixels belonging to $\mathcal {N}_k$, the 3D neighborhood of the *k*-th pixel. As example, a possible 26-pixels 3D neighborhood system is reported in Fig. 1.

The LGMRF model of Eq. (7) measures the differences between the value of each pixels and its surroundings, weighting them via the hyperparameters.

As it is clear from Fig. 1, not all voxels share the same distance from the considered one, as in general voxel spacing on the same slice is different from slice thickness, thus an additional spatial distance weighting term should be taken into account. Thus, the energy function of Eq. (7) is adapted, becoming:

8 $$\begin{aligned} E(b,\theta )=\sum _{k=1}^{U \times V \times S}\sum _{q\in {\mathcal {N}_k}} \frac{(b_k-b_q)^2}{2\theta _{k,q}^2 d_{k,q}^2} \end{aligned}$$

where the term $d_{k,q}$ is the euclidean distance between voxels $b_k$ and $b_q$.

From literature [20], the model is called “local” as the hyperparameters $\theta _{k,q}$ used for tuning the MRF are locally defined. A simple Gaussian MRF adopts a scalar hyperparameter (one value for the whole image [21]), while the LGRMF defines for each voxel multiple hyperparameters values (one for each neighboring location). The values $\theta _{k,q}$ allow to differently weight the neighboring pixels in the energy function. In particular, a low value of $\theta _{k,q}$ forces strong regularization since it is an index of high spatial correlation in the considered region (i.e. between $b_k$ and $b_q$). On the contrary, a high value of $\theta _{k,q}$ means low spatial correlation between pixels *k* and *q*, i.e. an edge or a small detail is more probable, lowering the filtering intensity. Clearly, the hyperparameters are not known and have to be estimated from the available data.

The maximum a posteriori estimator can be found by maximizing Eq. (1). After the application of a logarithmic transformation, the estimator of the noise free pixel intensity *b* can be equivalently written as:

9 $$\begin{aligned} \hat{b}= \arg \min _{b, b>0}\sum _{k=1}^{U \times V \times S} \Bigg \{ - \log \frac{(a_k - b_k)}{\sigma ^2} + \frac{\left( a_k - b_k \right) ^2}{2 \sigma ^2} + \sum _{q\in {\mathcal {N}_k}} \frac{(b_k-b_q)^2}{2\theta _{k,q}^2 d_{k,q}^2} \Bigg \} \end{aligned}$$

where $a_k$ is the measured pixel intensity at the location *k*. In Eq. (9), the first two addends within the curly brackets belong to the likelihood function of Eq. (5), while the last one is the a priori term derived from Eq. (8). From the above equation, it is evident that when the hyperparameters are high, the a priori energy tends to be small, producing a low regularized results. On the contrary, low hyperparameters, i.e. high spatial correlation, force the regularization of the image and thus a smooth solution. In this way, the filter behavior is locally adapted to the image, producing optimal results in terms of a posteriori distribution in an unsupervised way.

As initially the hyperparameters are unknown, the minimization of Eq. (9) is performed iteratively. Initially, hyperparameters $\theta$ are set equal to high values, in order to minimize the effect of the a priori information as the model has not already been tuned. At the end of each iteration, an estimation of *b* is computed for each voxel and exploited in order to update the hyperparameters $\theta$. Subsequently, Eq. (9) in minimized in order to achieve a new estimation of *b*. Thus, the process is iterated until convergence. If the 26-pixels 3D neighborhood is considered, within each iteration the hyperparameters are computed via:

10 $$\begin{aligned} \hat{\theta }^2_k=\frac{1}{26} \sum _{q\in {\mathcal {N}_k}} (\hat{b}_k-\hat{b}_q)^2 \end{aligned}$$

and the mean hyperparameter $\theta _{k,q}^2$ is computed as:

11 $$\begin{aligned} \hat{\theta }^2_{k,q}=\frac{1}{2} (\hat{\theta }^2_k+\hat{\theta }^2_q) \end{aligned}$$

**Fig. 2:**
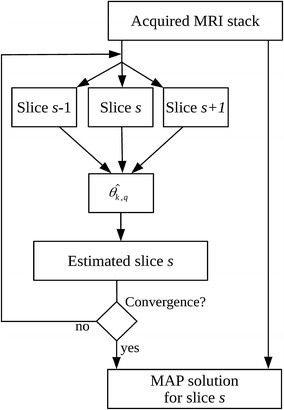
The processing chain of the proposed methodology. For each slice *s*, the 3D neighborhood is extracted by analyzing the upper ($s+1$) and lower ($s-1$) slices. The hyperparameters $\theta$ are evaluated and the estimation is performed. The procedure is iterated until convergence and repeated for all slices composing the 3D stack

In other words, once the data *a* are recorded, the noise free pixels *b* are estimated by computing cyclically Eqs.  (9), (10) and (11), until convergence. The processing chain of the algorithm is reported in Fig. 2. The stopping criteria is usually set by computing the mean value of the correction applied to image pixel *b*: when it decreases below a fixed threshold, the iterations are stopped. From literature, it is known that such procedure reaches convergence [20]. In case of the proposed methodology, few iterations are needed.

## Simulation and experiments

Within this Section, results on simulated and real case studies in order to validate the proposed methodology are reported. For each dataset, the proposed approach has been compared to some widely adopted noise filters, i. e. nonlinear anisotropic filter [25], 3D bilateral filter [26], linear minimum mean squared error (LMMSE) filter [27] and block-matching and 3D filtering (BM3D) algorithm, a methodology based on non-local mean approach [10]. In case of algorithms requiring the manual set of filtering parameters, the values adopted in the cited studies have been considered. Details about the algorithms are provided in Table 1.

**Table 1:** Specifications of filters used for comparison

Method	Experiment
Anisotropic diffusion	$n_{Iter}=4,~ \Delta _t=3/44,~k=70,~ c= 1/[1 + ( \ \nabla _I k)^2]$
3D bilateral	$\sigma _{x,y}=5,~ \sigma _z=5,~ \sigma _R=15,~ sam_S=5,~ sam_R=15$
LMMSE	Window size = $7 \times 7$
BM3D	$\sigma _{noise}$ has been provided

All the considered algorithms have been implemented in Mathworks™Matlab^®^ environment by using a Dell™Optiplex 990 workstation with Linux Debian as operative system.

First, a simulated case study is implemented in order to quantitatively evaluate noise removal effectiveness. In particular, mean square error (MSE) and Structural Similarity Index (SSIM) [28] are considered as quality indexes.

The simulated case study exploits Matlab^®^ 3D MRI head phantom, which is composed of 27 slices of $128 \times 128$ voxels. Rice distributed noise has been considered with different scale parameter $\sigma$ in order to evaluate performances under different levels of noise. In particular, the adopted values were between 0.5 and 8, corresponding to an SNR between 9 and 33 dB.

**Fig. 3:**
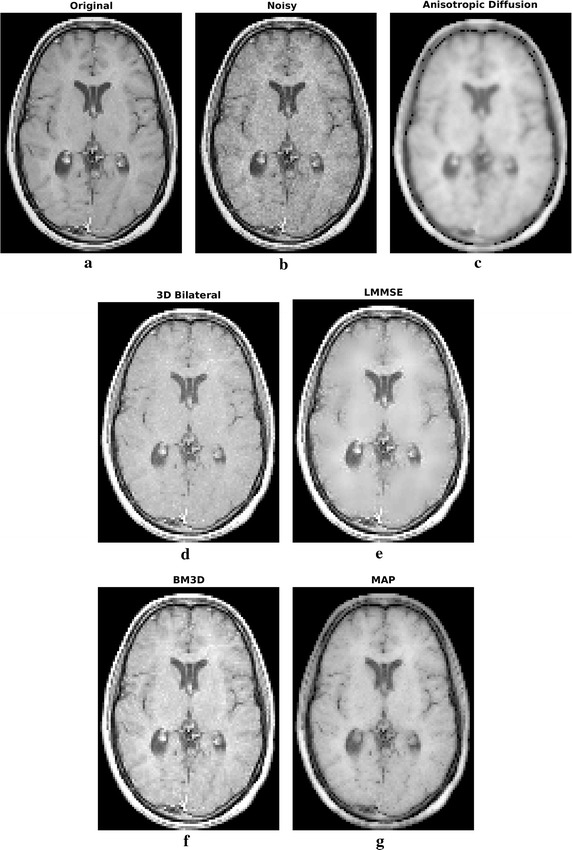
Filtering results for one slice of the phantom dataset: original (**a**) and noisy (**b**) image, filtering result achieved by anisotropic diffusion (**c**), 3D bilateral (**d**), LMMSE (**e**), BM3D (**f**) and proposed MAP (**g**) filters

**Fig. 4:**
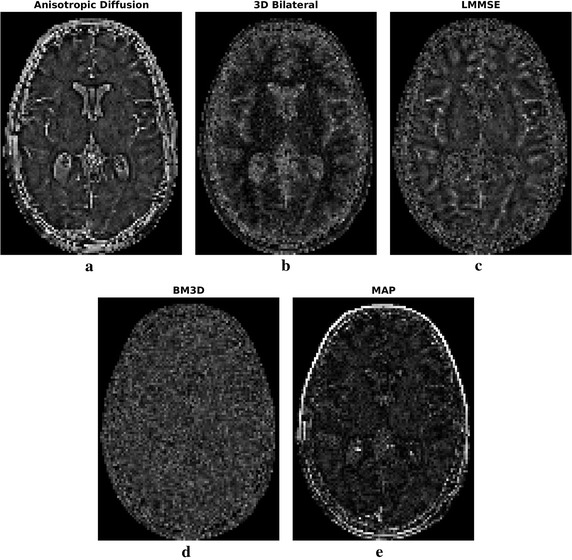
Residual noise for the considered slice of the simulated dataset. Proposed MAP approach solution has a residual noise lower than the other considered methods, with the exception of outer boundaries, where LGMRF model is less effective due to the lack of a complete neighborhood system

**Fig. 5:**
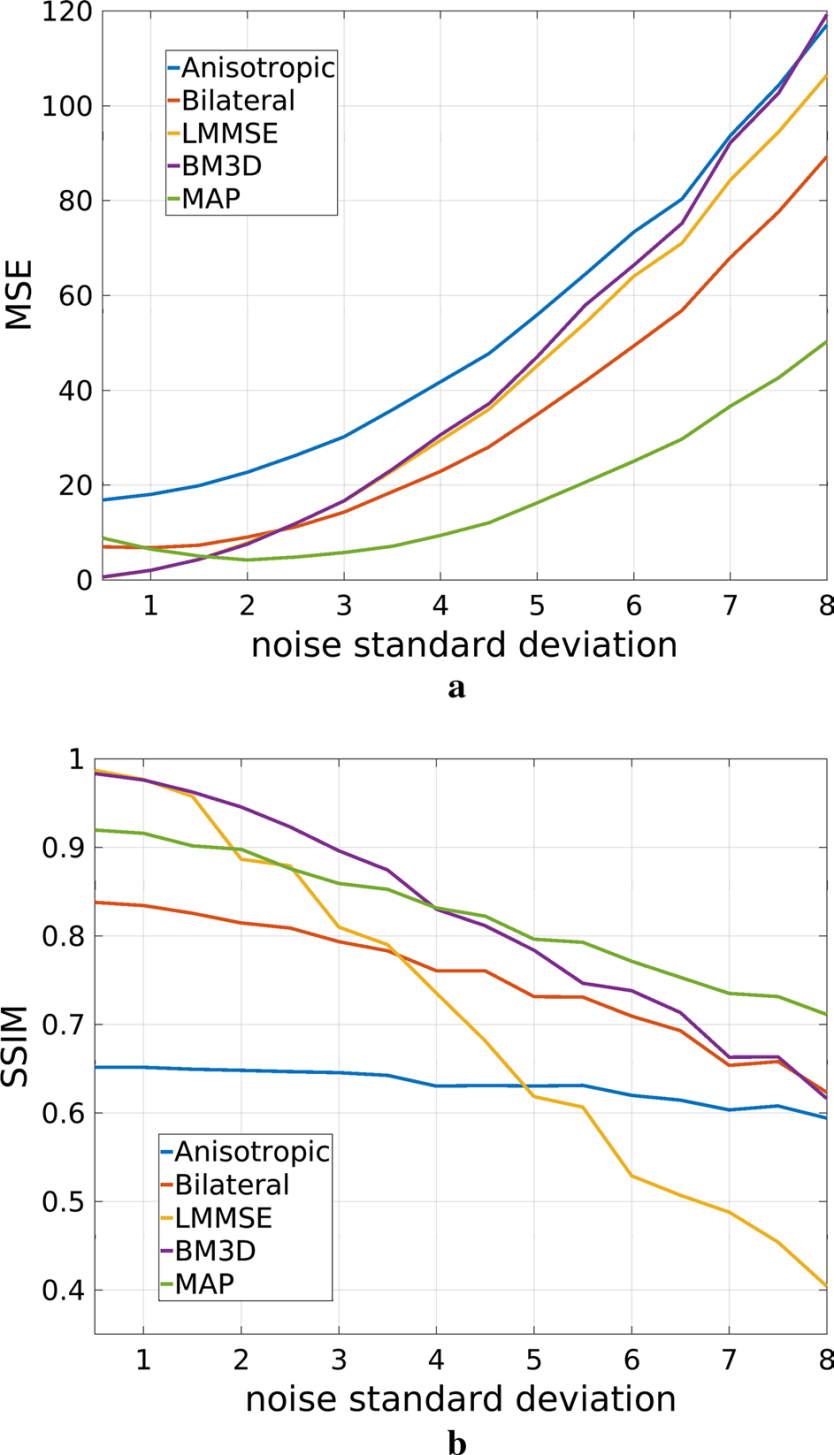
Mean square error (**a**) and Structural Similarity Index (**b**) computed for one slice of the considered phantom in case of different noise levels. In particular, noise standard deviation has been considered in the [0.5, 8] range, corresponding to an SNR between 33 and 9 dB

All the considered filters have been applied to the 3D stack, but in the following for simplicity only one of the slices (the central one) will be taken into consideration. However, it has to be underlined that filtering performances are substantially similar in case of the other slices. Results in case of $\sigma =4$ (SNR equal to 15 dB) are shown in Fig. 3, the residual noise, i.e. the difference between the images before and after filtering is reported in Fig. 4. The MSE and SSIM graphs, which have been computed in the brain region for all the algorithms, are plotted in Fig. 5.

Subsequently, three clinical datasets have been considered in order to qualitatively appreciate filters performances in case of real data. The first one refers to an MRI head acquisitions composed of 30 slices of $512 \times 512$ voxels acquired with a 3 T scanner at the IRCSS CROB hospital in Rionero in Vulture, Italy. The acquisition parameters are reported in Table 2. As the noise level is very low, the dataset has been corrupted by additive Rayleigh distributed noise in order to achieve an SNR equal to 10 dB. The informed consent was obtained from the involved subject.

**Table 2:** 3 T real dataset: imaging protocol details

MRI scanner	Philips achieva
Field intensity	3.0T
Sequence	Spin Echo
FOV	230 $\times$ 230 $\times$ 135 mm
Voxel size	0.45 $\times$ 0.45 $\times$ 4.5 mm
Stack resolution	512 $\times$ 512 $\times$ 30 pixels

The second real dataset also refers to an MRI head axial acquisition, but an 1.5 T scanner was considered. This dataset is public available on the dicom.nema.org website. The acquisition parameters are reported in Table 3.

**Fig. 6:**
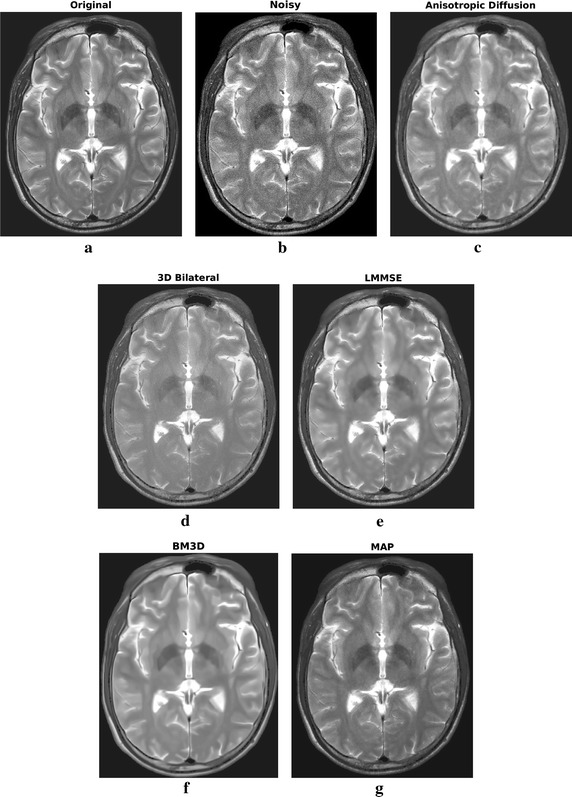
Filtering results for one slice of the 3 T real dataset: acquired image (**a**), noisy one (**b**), filtering result achieved by anisotropic diffusion (**c**), 3D bilateral (**d**), LMMSE (**e**), BM3D (**f**) and proposed MAP (**g**) filters

**Fig. 7:**
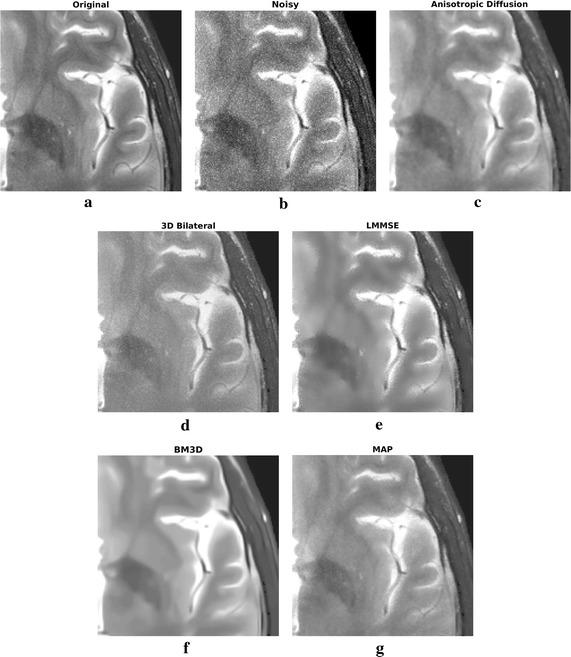
Filtering results for one slice of the 3 T real dataset: enlargement over a ROI

**Fig. 8:**
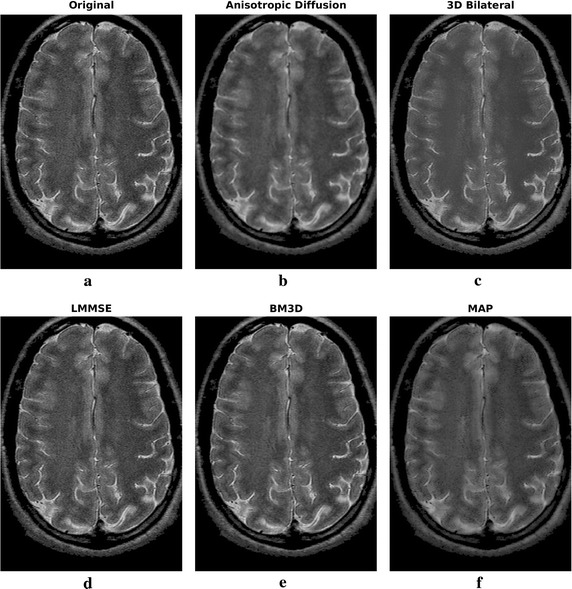
Filtering results for one slice of the 1.5 T real axial dataset: acquired noisy image (**a**), filtering result achieved by anisotropic diffusion (**b**), 3D bilateral (**c**), LMMSE (**d**), BM3D (**e**) and proposed MAP (**f**) filters

**Fig. 9:**
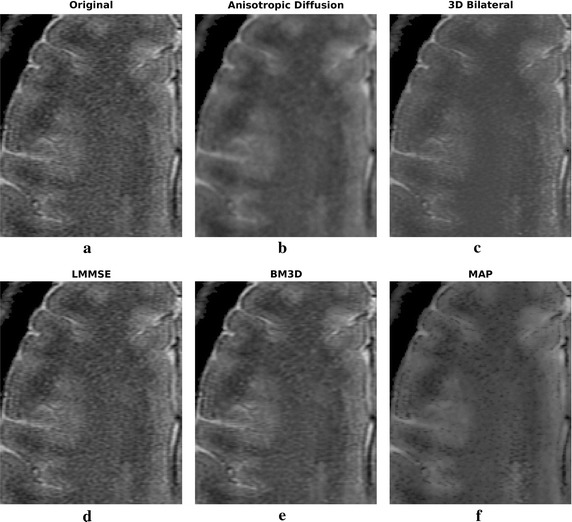
Filtering results for one slice of the 1.5 T real axial dataset: enlargement over ROI 1

**Fig. 10:**
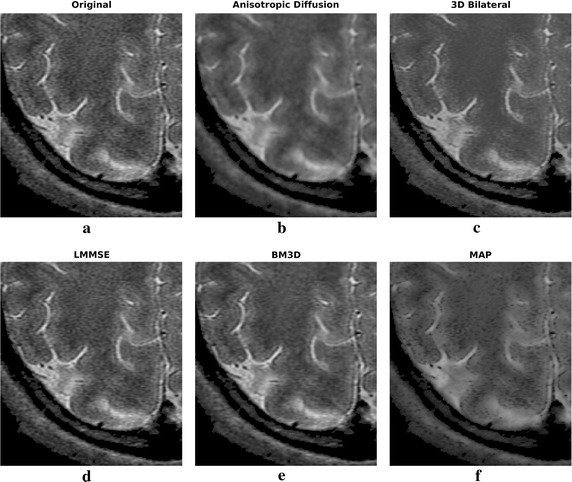
Filtering results for one slice of the 1.5 T real axial dataset: enlargement over ROI 2

Filtering results of all the considered algorithms for the clinical datasets are reported in Figs. 6 and 8, respectively, while enlargements can be found in Figs. 7, 9 and 10.

The third real dataset refers to an MRI sagittal acquisition of the vertical column in the lumbar area, again acquired with a 1.5 T scanner. Also this dataset is public available on the http://www.osirix-viewer.com website. The acquisition parameters are reported in Table 4.

**Table 3:** 1.5 T real axial dataset: imaging protocol details

Field intensity	1.5 T
Sequence	Fast Spin Echo
Modality	T2 weighted
FOV	220 $\times$ 220 $\times$ 128 mm
Voxel size	0.43 $\times$ 0.43 $\times$ 2.0 mm
Stack resolution	512 $\times$ 512 $\times$ 64 pixels

**Fig. 11:**
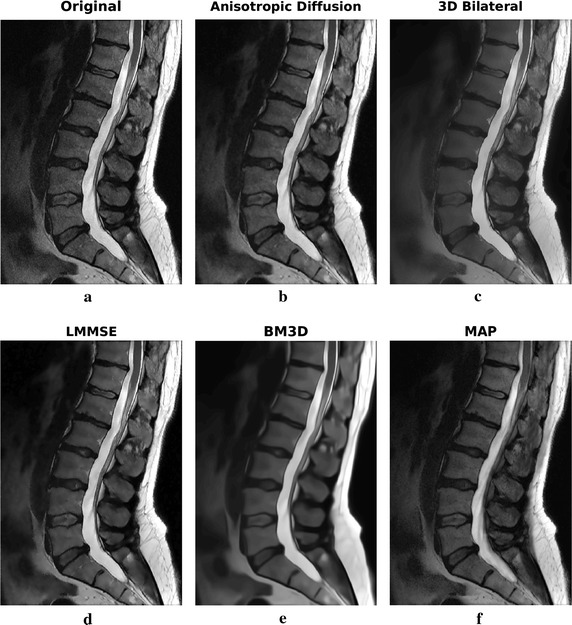
Filtering results for slice 5 of the 1.5 T real sagittal dataset: acquired noisy image (**a**), filtering result achieved by anisotropic diffusion (**b**), 3D bilateral (**c**), LMMSE (**d**), BM3D (**e**) and proposed MAP (**f**) filters

**Fig. 12:**
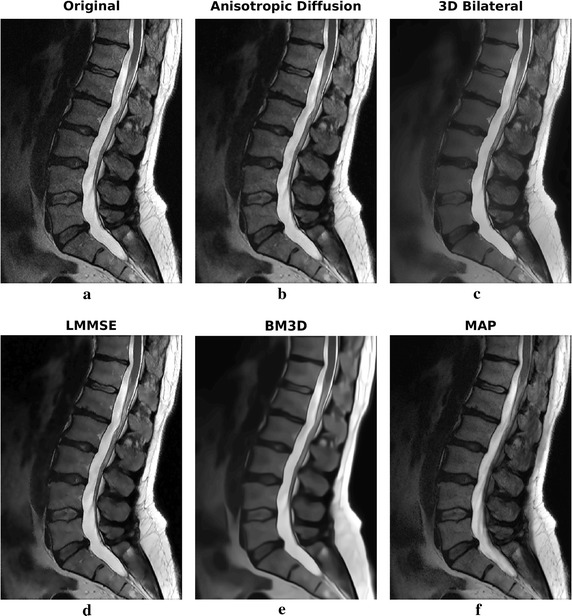
Filtering results for slice 6 of the 1.5 T real sagittal dataset: acquired noisy image (**a**), filtering result achieved by anisotropic diffusion (**b**), 3D bilateral (**c**), LMMSE (**d**), BM3D (**e**) and proposed MAP (**f**) filters

**Fig. 13:**
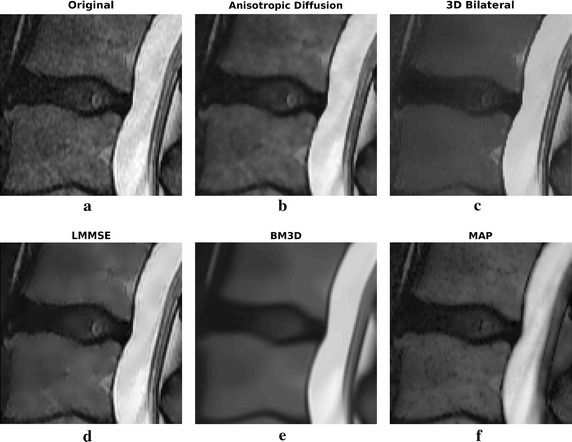
Filtering results for slice 6 of the 1.5 T real sagittal dataset: enlargement over ROI 1

Two slices of the sagittal dataset have been considered. The filtering results are reported in Figs. 11 and 12. Enlargements over one ROI are reported in Fig. 13.

In order to give an idea of the computational load, the filtering of this dataset took about 130 s/iteration on an Intel Core i7 workstation, while anisotropic diffusion, 3D bilateral, LMMSE and BM3D completed the processing in 6, 0.5, 0.2 and 2 s, respectively. The computational complexity of the MAP approach is mainly due to the computation of the hyperparameter for each couple of neighboring pixels. However, it has to be underlined that the MAP code has been developed in Matlab^©^ environment, and no code optimization was done, differently from the other considered algorithms. It is expected a two order of magnitude speedup can be achieved by paralleling and optimizing the code.

**Table 4:** 1.5 T real sagittal dataset: imaging protocol details

Field intensity	1.5 T
Sequence	Fast Spin Echo
Modality	T2 weighted
FOV	281 $\times$ 281 $\times$ 45 mm
Voxel size	0.55 $\times$ 0.55 $\times$ 5.0 mm
Stack resolution	512 $\times$ 512 $\times$ 9 pixels

## Results and discussion

By looking at the filtering results in case of the simulated case study, reported in Fig. 3, qualitative information on the algorithm filtering effectiveness can be inferred. The anisotropic diffusion filter produces the smoothest results, with very low performances in terms of edges and details retrieval. Also the LMMSE filtered image is blurred, with constant areas and very few small anatomical structures visible. On the other hand, 3D bilateral and BM3D approaches are capable of better details preservation, but the noise reduction effectiveness is limited. Proposed MAP approach can be placed in the middle. The preservation accuracy of edges and small elements is globally similar to BM3D filter, but the noise reduction effectiveness is higher.

Both SSIM and MSE graphs computed for different noise levels and reported in Fig.  5 confirm such findings. In particular, the MSE curves show a higher effectiveness of proposed MAP approach in reducing noise, with a small advantage of BM3D only in case of very low noise ($\sigma \le 1.5$, SNR $\ge$ 23 dB). We recall that the proposed MAP methodology has been developed under the assumption of low SNR, so an MSE deterioration in case of low $\sigma$ (not noisy) is expected.

Moving to the SSIM index, which is mostly related to details preservation, MAP and BM3D approaches are characterized by similar performances, with an advantage of the former in case of strong noise ($\sigma \ge 5$, SNR $\le$ 13 dB) and of the latter in case of weak noise ($\sigma \le 3$, SNR $\ge$ 17 dB), as expected again.

By looking at the residual error maps of Fig. 4, which refer to the $\sigma =4$ case, different behaviors of the considered approaches can be found. In particular, the anisotropic diffusion and the LMMSE filters show a residual error mostly concentrated on edges, indicating poor detail preservation capability and confirming the low values of SIM reported in Fig. 5. The 3D bilateral and the MAP filters, show small errors across the whole image (smooth areas and details). Visually, the errors of the proposed MAP approach are lower than the 3D bilateral filter, as expected from the MSE values. Moreover, they are mainly concentrated on the outer edges: this result was expected, because of the lack of a useful neighborhood on the external edges. The BM3D residual error lays in the middle of the two categories, as the error intensity is not low but at the same time it is not concentrated close to the edges.

Moving to the 3 T real dataset, let us focus on Fig. 6 and on the enlargements over a region of interest (ROI) placed in the top right corner, reported in Fig. 7. As in the simulated case study, the LMMSE filter over-smooths the images, loosing many details, while the 3D bilateral approach is not very effective in reducing noise. Concerning the anisotropic diffusion filter, interesting results both in terms of noise reduction and edges preservation are achieved. This is in contrast with the poor results of the simulated dataset previously reported. Such behavior can be explained considering the high supervision required by the approach: the same configuration parameters (the default ones) have been adopted for both real and simulated test cases, providing results with very different accuracy in the two case studies. This aspect greatly influences the applicability of the Anisotropic Diffusion filter. The BM3D algorithm result is very clean, and noise reduction is considerable, although its behavior is opposite with respect to anisotropic diffusion: the good results of the simulated dataset are not confirmed. As in the previous case, the effect is probably due to the several configuration parameters required by the algorithm. We underline that the default parameters adopted by the authors in [10] were considered in this manuscript.

Moving to the proposed MAP approach, again the filtered image is a good compromise between noise reduction and details preservation, providing good reconstructions without requiring any parameter setting. In particular, looking at the enlargement reported in Fig. 7, it is clear the strong smoothing of LMMSE and BM3D approaches, and the good details preservation of anisotropic diffusion, 3D bilateral and MAP approaches, with the latter appearing a good trade-off between smoothness and sharpness.

The real 1.5 T case axial study mainly confirms previous results. With respect to 3 T case, a higher noise is corrupting the images. The LMMSE in this case fails in detecting the smooth areas, producing a very poor regularization effectiveness. That said, again the performances of 3D bilateral and proposed MAP approaches are similar, with slight higher noise removal effectiveness for the latter. anisotropic diffusion solution is very smooth, while BM3D approach produces a result that is in between LMMSE and 3D bilateral in terms of details preservation and noise smoothing. In the enlargements reported in Figs. 9 and 10 it is evident that MAP is also effective in handling smooth transition, with the boundary between white matter and gray matter that is well recognized. Moreover, a lot of small details are correctly retrieved in the filtered image.

Moving to the second 1.5 T dataset, the sagittal acquisition of a portion of the vertebral column, results over two consecutive slices are reported. LMMSE and BM3D algorithms produce an over-smoothed solution, with some fine details lost across the image. On the other side, The anisotropic diffusion approach preserves a lot of details at the cost of not very effective noise removal. Among all, the 3D bilateral approach produces the most similar results with respect to the MAP algorithm. Moving to the ROI of Fig. 13, it allows to better appreciate the differences among algorithms. In particular, it is evident that only the proposed MAP filter is able to correctly retrieve the spinal cord texture.

## Conclusions

Within this manuscript, a denoising methodology for MR images has been presented. The algorithm implements a maximum a posteriori (MAP) estimator and models the 3D acquired data via a local Gaussian Markov random field (LGMRF). The peculiarity of LGMRF consists in its ability to adapt itself to the local behavior of the imaged slices, allowing the preservation of small anatomical structures and edges filtering without any supervision. Simulated and real data results show that, compared to other widely adopted MRI denoising algorithm such as linear minimum mean square error (LMMSE), block-matching and 3D (BM3D), 3D bilateral and nonlinear anisotropic diffusion filters, a good noise removal effectiveness is achieved by MAP together with interesting performances in terms of edges and details preservation. In particular, compared to 3D bilateral and BM3D filters it showed similar edge preservation effectiveness, but with higher regularization capability in smooth areas without manual set of any filter parameter.

The achieved results together with its unsupervised nature potentially make the proposed approach an interesting and promising instrument for denoising clinical images.

Issues regarding some implementation aspects of the proposed methodology are still open, and will be addressed in the future. In particular, further studies on the optimal size of the neighborhood system and on the improvement of the computational efficiency of the filter.

## Abbreviations

BM3D: block-matching and 3D filtering
DT: diffusion tensor
LGMRF: local Gaussian MRF
LMMSE: linear minimum mean squared error
MAP: maximum a posteriori
MLE: maximum likelihood estimator
MR: magnetic resonance
MRF: Markov random field
MRI: magnetic resonance imaging
MSE: minimum square error
ROI: region of interest
SNR: signal to noise ratio
SSIM: Structural Similarity Index
